# T_1_ based oxygen-enhanced MRI in tumours; a scoping review of current research

**DOI:** 10.1259/bjr.20220624

**Published:** 2023-03-03

**Authors:** Alastair McCabe, Stewart Martin, Jagrit Shah, Paul S Morgan, Rafal Panek

**Affiliations:** 1 Academic Unit of Translational Medical Sciences, School of Medicine, University of Nottingham, Nottingham, United Kingdom; 2 Department of Clinical Oncology, Nottingham University Hospitals NHS Trust, Nottingham, United Kingdom; 3 Department of Radiology, Nottingham University Hospitals NHS Trust, Nottingham, United Kingdom; 4 Mental Health & Clinical Neurosciences Unit, School of Medicine, University of Nottingham, Nottingham, United Kingdom; 5 Department of Medical Physics & Clinical Engineering, Nottingham University Hospitals NHS Trust, Nottingham, United Kingdom

## Abstract

**Objective:**

Oxygen-enhanced MRI (OE-MRI) or tissue oxygen-level dependent (TOLD) MRI is an imaging technique under investigation for its ability to quantify and map oxygen distributions within tumours. The aim of this study was to identify and characterise the research into OE-MRI for characterising hypoxia in solid tumours.

**Methods:**

A scoping review of published literature was performed on the PubMed and Web of Science databases for articles published before 27 May 2022. Studies imaging solid tumours using proton-MRI to measure oxygen-induced T_1_/R_1_ relaxation time/rate changes were included. Grey literature was searched from conference abstracts and active clinical trials.

**Results:**

49 unique records met the inclusion criteria consisting of 34 journal articles and 15 conference abstracts. The majority of articles were pre-clinical studies (31 articles) with 15 human only studies. Pre-clinical studies in a range of tumour types demonstrated consistent correlation of OE-MRI with alternative hypoxia measurements. No clear consensus on optimal acquisition technique or analysis methodology was found. No prospective, adequately powered, multicentre clinical studies relating OE-MRI hypoxia markers to patient outcomes were identified.

**Conclusion:**

There is good pre-clinical evidence of the utility of OE-MRI in tumour hypoxia assessment; however, there are significant gaps in clinical research that need to be addressed to develop OE-MRI into a clinically applicable tumour hypoxia imaging technique.

**Advances in knowledge:**

The evidence base of OE-MRI in tumour hypoxia assessment is presented along with a summary of the research gaps to be addressed to transform OE-MRI derived parameters into tumour hypoxia biomarkers.

## Introduction

The presence of biologically significant hypoxia in tumours has been known since the work of Thomlinson and Gray in the 1950’s.^1^ More recent studies show that the presence of oxygen concentrations below 10 mmHg in tumours results in significantly worse radiotherapy and chemotherapy treatment outcomes as well as increasing the risk of distant metastases.^2,3^ The accurate and reliable assessment of tumour hypoxia is important in identifying tumours more likely to respond to hypoxia modifying treatments and in monitoring the effect of such interventions. In addition, mapping the spatial distribution of hypoxic regions would allow targeting of treatment resistant areas with increased radiotherapy doses. Consequently, there has been significant interest in hypoxia imaging.

**Figure 1.**
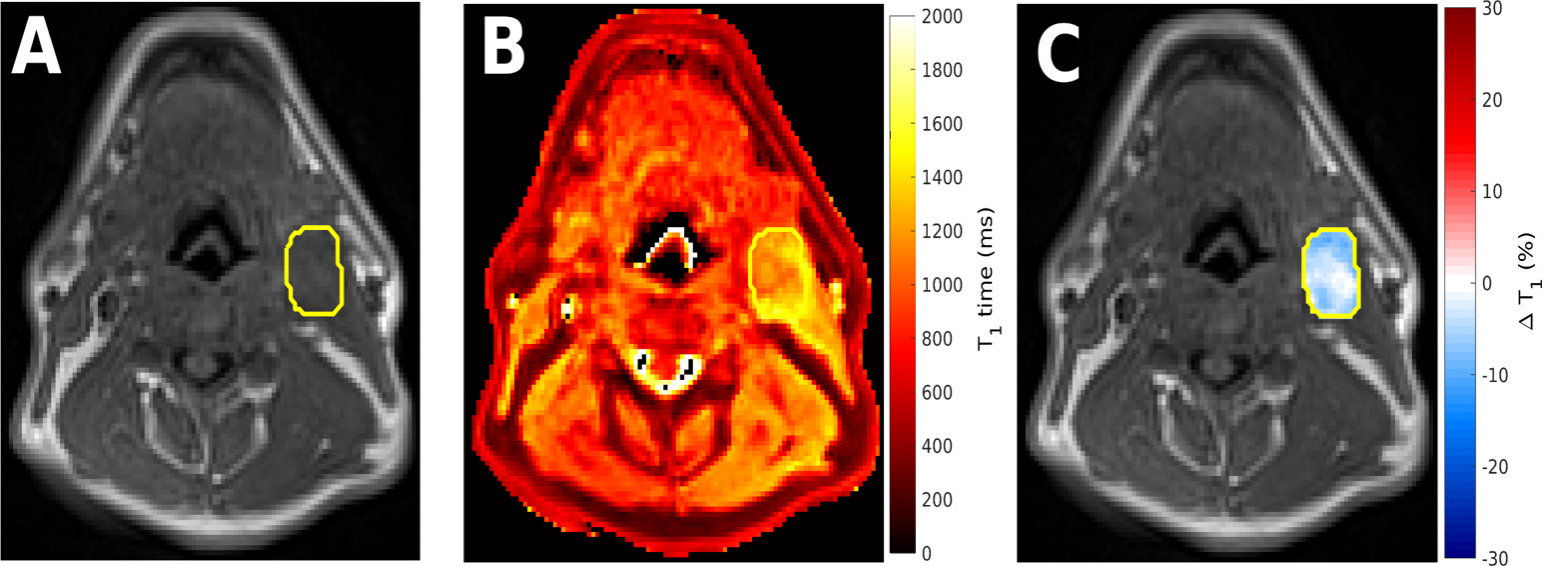
Example images from a patient with head and neck squamous cell carcinoma illustrating the difference between conventional *T*_1_ weighted imaging, quantitative *T*_1_ mapping and oxygen induced quantitative Δ*T*_1_ maps. (A) *T*_1_ weighted anatomical image (3D SPGR, TR/TE = 10/1.27 ms, FA = 18°). (B) Corresponding quantitative *T*_1_ map derived using the variable flip angle methodology from image A and a corresponding proton density weighted image (not shown, acquired with same parameters but FA = 2°). (C) Oxygen induced Δ*T*_1_ map obtained following a supplemental oxygen challenge administered using 15 L/min oxygen via a non-rebreather mask. Within the highlighted malignant nodal mass, regions of oxygen induced *T*_1_ shortening implying normoxia are clearly seen. Distinct oxygen refractory regions are also identifiable which imply either hypoxic or non-perfused areas. FA, flip angle; TE, echo time; TR, repetition time.

An emerging MRI-based technique to map hypoxia is oxygen-enhanced MRI (OE-MRI) or tissue oxygen level dependent (TOLD) MRI which only requires routinely available healthcare equipment; namely an MRI scanner and oxygen delivery mechanism. OE-MRI relies on the weakly paramagnetic property of molecular oxygen due to the presence of unpaired electrons. Elevated tissue concentrations of dissolved oxygen increase tissue’s longitudinal relaxation rate (R_1_ = 1 /T_1_ where T_1_ is the longitudinal relaxation time).^4,5^ Following the delivery of supplemental oxygen (known as an oxygen challenge), initially well-oxygenated regions where haemoglobin is well saturated develop an increase in dissolved molecular oxygen with consequential reduction in T_1_ times. However in perfused hypoxic regions where haemoglobin is less well saturated, administration of high concentration oxygen leads to an increase in oxyhaemoglobin over dissolved molecular oxygen meaning tissue T_1_ times do not shorten. By administering high concentration oxygen and performing T_1_ mapping, areas of hypoxia can be identified from a lack of decrease in T_1_ times (Figure 1).

The purpose of this scoping review is to review the current evidence for MRI of hypoxia in solid tumours using a supplemental oxygen challenge to induce changes in proton T_1_ relaxation rates and identify areas requiring further research in order to translate OE-MRI into a routinely used clinical technique.

## Methods

This scoping review follows the PRISMA extension for scoping reviews (PRISMA-ScR)^6^ but was not registered on an online database. The literature search was performed on 27 May 2022 on the PubMed and Web of Science databases using identical search strategies (Appendix 1). There were no limits on publication dates.

Supplementary Material 1.

Results from the searches were combined, duplicates removed and articles restricted to novel research. References from excluded review articles were manually examined for additional resources. Two independent reviewers screened the articles against the following inclusion criteria:

1. Images solid tumours in animal models and/or human participants
2. Uses proton-based MRI.
3. Assesses changes in T_1_ relaxation times (or R_1_ relaxation rates, R_1_ = 1/T_1_) following a supplemental oxygen challenge

Discrepancies between reviewers were discussed and consensus reached. Grey literature was searched by interrogating abstracts from the International Society of Magnetic Resonance in Medicine annual meeting from 2011 to 2022 and the ClinicalTrials.gov website for currently open trials satisfying the eligibility criteria.

To examine the full scope of OE-MRI in solid tumours, we included pre-clinical and clinical research and did not restrict our results to a particular tumour subtype. As such, we expected significant heterogeneity in the results and relatively few publications in any one tumour type. We therefore did not plan to perform any quantitative analysis and did not have pre-defined critical appraisal criteria as we did not want to reject studies at this stage. We planned to present our findings in the form of a descriptive review.

## Results

**Figure 2.**
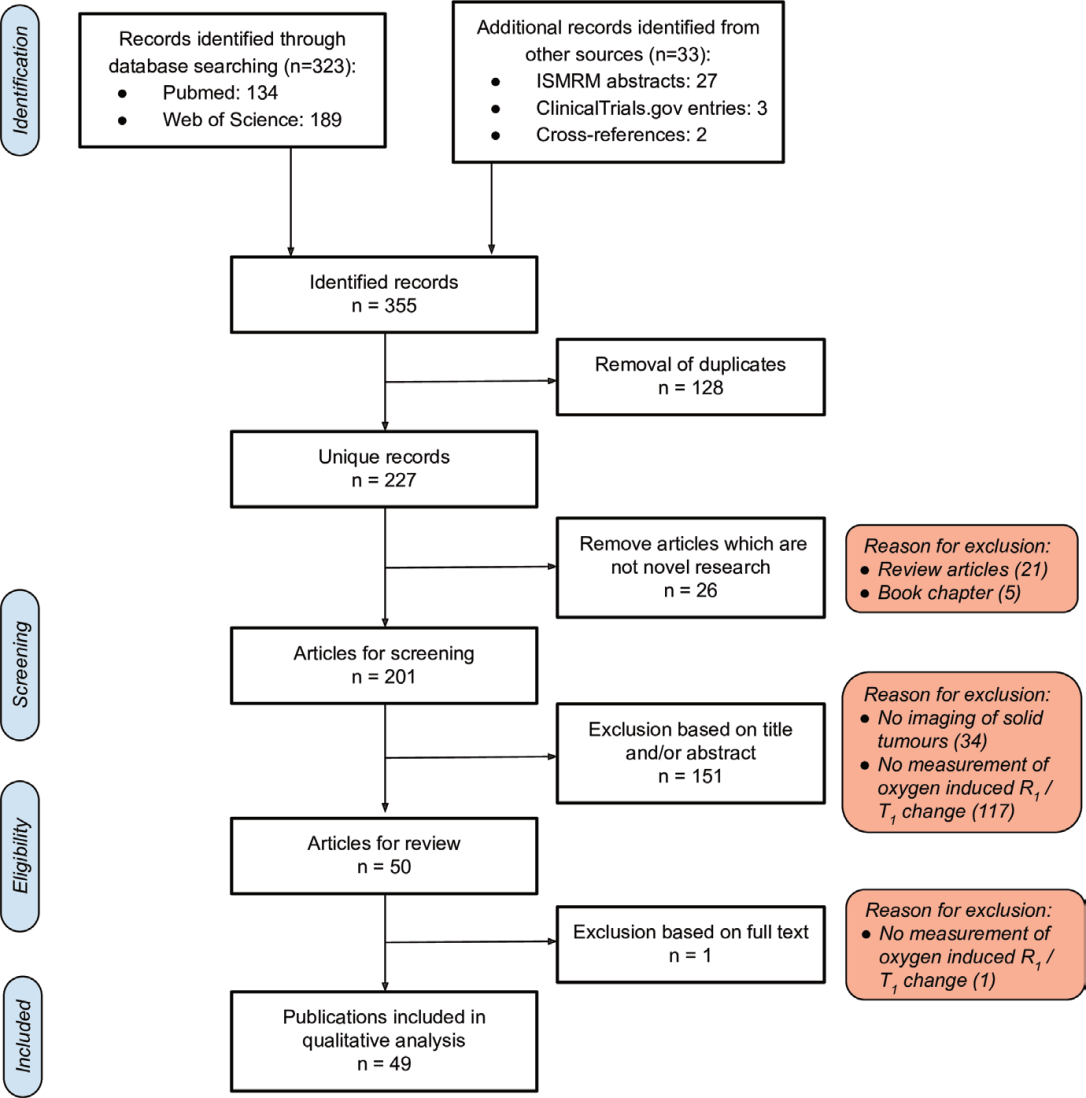
Flowchart showing the results of the search strategy.

**Figure 3.**
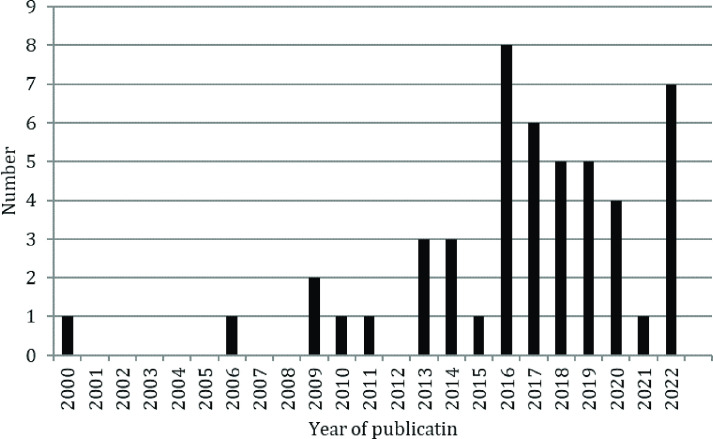
Histogram of publication year for journal article articles and conference abstracts identified in the search.

227 unique records were identified. Following screening, 49 articles were identified for qualitative analysis consisting of 34 journal articles and15 conference abstracts (Figure 2, full list of results in Appendix 2). The distribution of the journal articles and published conference abstracts by publication year is shown in Figure 3. Three trials satisfying the eligibility criteria that were listed as open to recruitment were identified on the ClinicalTrials.gov website.^7–9^ All of these studies have corresponding conference abstracts included in the final search results.^10–12^

Supplementary Material 2.

### Research focus

The majority of published studies involve animal only research (31 studies, 63.3%) with 15 being human only studies (30.6%) and 3 being mixed studies (6.1%). Of those involving human participants, 4 image intracranial neoplasms and 4 scan head and neck malignancies (22.2% each). 2 references image colorectal cancer and hepatocellular cancer (11.1% each) with the remaining results divided between single studies in lung, anal, cervical, renal, prostate and mixed tumour sites.

### Pre-clinical studies

Four main associations have been tested in pre-clinical models to validate the ability of OE-MRI in detecting tumour hypoxia:

1. Validation against alternative oxygenation determining techniques including direct measurements and alternative imaging strategies such as hypoxia PET scanning.^13–18^
2. Verification that the distribution of hypoxic and normoxic areas seen with OE-MRI display intratumoural heterogeneity but with spatially coherent regions in keeping with patterns known to occur biologically.^5,17–35^
3. Correlation against histopathological hypoxia indicators such as pimonidazole staining, glucose transporter 1 (GLUT-1) expression and hypoxia-inducible factor 1-α (HIF-1α) expression.^17,29,31,32,34–38^
4. Verification of OE-MRI’s ability to predict tumours more likely to display hypoxia induced treatment resistance. ^16,31,39–41^

One of the papers that helped establish the utility of OE-MRI in accurately mapping tumour hypoxia was based on renal and colorectal carcinoma cell lines implanted in mice.^17^ The authors correlated OE-MRI data with direct measurements of tissue oxygen concentration and established a correlation between OE-MRI quantified tumour hypoxic fraction and histopathological staining with the hypoxia sensitive marker pimonidazole. The authors also demonstrated that OE-MRI could detect expected increases in tumour hypoxic fractions following administration of the vasodilator drug hydralazine.^17^ A separate study in human lung adenocarcinoma xenografts helped biologically validate the OE-MRI technique by demonstrating its ability to detect changes in tumour hypoxic volumes following administration of a bioreductivecytotoxin (banoxantrone) and an oxygen consumption modifier (atovaquone).^40^

The original OE-MRI analysis method calculates the spatial average of T_1_/R_1_ changes with oxygen over the imaged tumour. However, a number of papers fail to show correlations of these spatially averaged values with reference hypoxia markers^17,24,29,42^ or tumour radiosensitivity indicators^25,31,43^ possibly due to the heterogeneous distributions of oxygen within tumours resulting in hypoxic regions being masked by the T_1_/R_1_ changes induced in normoxic areas. O’Connor et al.proposed combining OE-MRI with a perfusion assessment thereby enabling voxels of interest to be identified as perfused oxygen-enhancing (normoxia), perfused oxygen-resistant (hypoxia) or nonperfused (necrosis).^17^ The perfused oxygen-resistant biomarker is sensitive to spatial fluctuations in hypoxia and shows correlation with alternative hypoxic markers and clinically relevant tumour hypoxia outcomes in both pre-clinical^17,29,31^ and human studies.^29,31,44,45^ Such perfusion masks have been generated using dynamic contrast-enhanced (DCE) MRI^17,29,31,44,45^ and ultrasmall superparamagnetic iron oxide (USPIO)-enhanced MRI derived fractional blood volume measurements.^32^

An alternative OE-MRI approach combining R_2_* (Blood Oxygen Level Dependent (BOLD)) and R_1_-based oxygen-enhanced imaging aims to distinguish blood-based oxygen-induced changes from tissue-based ones. Although increased concentration of dissolved oxygen accelerates R_1_ relaxation rates, the net tissue R_1_ rate is affected by a number of other factors including being reduced by lower concentrations of deoxyhaemoglobin.^46^ Although this influence is generally small compared to the influence of dissolved oxygen, the simultaneous measurement of R_2_* values may yield insights into the cause of observed oxygen-induced tissue R_1_ decreases.^20,25,36,47,48^ In particular, Cao-Pham et al present a hypothesis based on their work on rhabdomyosarcoma and glioma xenografts where they divide voxels into four classes dependent upon the relative changes in oxygen induced R_1_ and R_2_* rates^25^:

1. *Normoxia:* Significantly increasing R_1_ (increased molecular oxygen) and stable or mildly increased R_2_* (stable deoxy/oxyhaemoglobin ratio)
2. *Mild hypoxia:* Slightly increasing R_1_ (increased molecular oxygen) with decreasing R_2_* (decreased deoxy/oxyhaemoglobin ratio). Assumes unsaturated baseline haemoglobin changing to near complete saturation.
3. *Severe hypoxia*: No/mild decreasing R_1_ and decreasing R_2_* (decreased deoxy/oxyhaemoglobin ratio)
4. *Vascular Steal:* Decreasing R_1_ with increasing R_2_*. Hypothesised to be caused by dilatation of mature blood vessels shunting blood away from tumour regions served by immature vessels resulting in decreased blood volume and molecular oxygen.

The use of a cyclical oxygen challenge combined with independent component analysis of the voxel-wise signal traces has been proposed as another method to improve the sensitivity of OE-MRI. Hypoxic regions derived using this approach have been correlated to pimonidazole stained areas in murine squamous cell carcinomas^38^ and shown capable of detecting oxygenation changes in murine tumours following treatment with vascular growth factor inhibition.^30^ However, poor correlation with human colorectal xenografts was noted possibly due to the lack of a perfusion assessment meaning that regions of necrosis may have confounded the imaging assessment.^38^

Regarding correlations of OE-MRI parameters with pre-clinical treatment outcomes, 3 studies in prostate cancer found oxygen-induced R_1_ changes correlated with outcomes following radiotherapy^16,39,41^ but 1 paper found no association between OE-MRI parameters and local tumour control probability.^43^ This study used tumour mean oxygen-induced R_1_ changes rather than the perfused fraction biomarker. Salem et al found that OE-MRI determined biomarkers detected therapy-induced changes in hypoxia in glioma xenografts and non-small cell lung cancer (NSCLC)^31^ but all studies that have looked at correlating OE-MRI biomarkers with treatment outcomes have been with relatively small study sizes.

### Human studies

OE-MRI studies on human participants have been performed on all major anatomical regions with no significant difficulties reported with patient tolerability. The principle research foci of the human studies are shown in Table 1 categorised by domains derived from the Cancer Research UK and European Organisation for Research and Treatment of Cancer consensus statement on the clinical translation of imaging biomarkers.^49^

**Table 1.** Summary of principle research focus of tumour OE-MRI studies in humans categorised by domains derived from the CRUK and EORTC consensus statement on the clinical translation of imaging biomarkers^49^

Clinical research focus	Number of studies	References
Proof of principle including safety, feasibility and tolerability	15	^5,10–12,21,31,36,44,45,50–55^
Repeatability and reproducibility	5	^31,44,50,54,55^
Correlation with histopathology	2	^29,56^
Changes of biomarkers with treatment	7	^11,31,45,52,53,55,57^
Initial correlation of biomarkers to clinical outcomes	1	^52^
Prospective, adequately powered studies linking biomarkers to clinical outcomes	0	N/A
Analysis techniques	7	^5,21,28,29,31,36,58^
Multicentre studies	0	N/A
Health economic considerations	0	N/A

CRUK, Cancer Research UK; EORTC, European Organisation for Research and Treatment of Cancer.

Three studies performed OE-MRI assessments in both pre-clinical and clinical settings and all demonstrate similar patterns between animal and human scans.^29,31,36^ The studies investigating changes of OE-MRI biomarkers with treatment in patients have been performed in glioblastoma, ^57^ brain metastases,^53^ head and neck cancer,^55^ NSCLC,^31^ cervical cancer,^11^ rectal cancer^45^ and anal cancer.^52^

Salem et al used the perfused hypoxic fraction metric in patients with NSCLC to distinguish tumours that have persistent hypoxia from those that demonstrate hypoxia modification with chemoradiotherapy.^31^ Similarly, Little et al scanned patients with rectal cancer immediately prior to chemoradiotherapy and again at day 7 or day 14 of treatment and found measurable changes in tumour hypoxic burden with treatment using the perfused Oxy-R metric. Reduction in tumour hypoxia was only apparent by day 14 though and not at day 7.^45^ Reduction in hypoxic fractions with treatment is also found in the anal, cervical and head and neck cancer studies with the latter additionally demonstrating the feasibility of performing OE-MRI on the MR-Linac (hybrid MRI scanner and radiotherapy linear accelerator).^11,52,55^

### Technical considerations

A range of methodologies have been used in clinical OE-MRI research (Tables 2 and 3). Human studies show a preference for 1.5 T (11 studies, 61.1%) over 3 T (9 studies, 50.0%) imaging systems (2 studies utilised both field strengths). The most frequently used T_1_ measurement technique in human OE-MRI is the variable flip angle (VFA) method, used by 50% of studies, closely followed by inversion recovery-based techniques. An alternative T_1_ mapping technique called MOBILE (mapping of oxygen by imaging lipids relaxation enhancement) that exploits the increased solubility of oxygen in lipids over water has also been investigated.^14^ Studies in tumour models found that oxygen induced changes in lipid R_1_ rates were of greater magnitude than changes in water R_1_ and global R_1_rates.^14,15^ This approach however might be sensitive to the amount of lipid present within tumours.^42^

**Table 2.** Imaging parameters and setup details for the T_1_-based oxygen-enhanced MR scans for the peer-review published in human studies

	O’Connor et al. 2009^5^	Remmele et al. 2013^21^	Linnik et al. 2014^36^	Bane et al. 2016^49^	Hectors et al. 2017 a^55^	Hectors et al. 2017 b^55^	Zhou, Hallac. et al. 2017^50^	Little et al. 2018^29^	Salem et al. 2019^31^	Bluemke et al. 2020^51^	Qian et al. 2020^52^	Bluemke et al. 2022 a^10^	Bluemke et al. 2022 b^10^
Area	Liver, omentum, pelvis	Brain	Brain	Liver	Liver	Liver	Prostate	Kidney	Lung	Anus	Brain	Head and neck	Head and neck
B0	1.5T	3T	3T	1.5T	3T	1.5T (3T)	3T	1.5T	1.5T	3T	3T	3T	3T
Sequence	3D FFE	2D-SPGR simultaneous R1/R2*	3D FFE	IR-LL	3D VFA	2D IR-LL	FFE	IR	IR-prepared3D-SPGR	MOLLI	FFE	MOLLI	IR-prepared3D-SPGR
TR / TE (ms)	3.5/0.9	103/12 echoes	3.5/1.1	2.26/1.04	10/1.2	2.3/1.0 (35.1/1.2)	-	10,000/3.1	2.1/0.5	3,000/120	32/2.0	3.05/1.332	4/0.656
TI (ms)	-	-	-	42–1576.5	-	42-1577 (80–1445)	-	50, 200, 500, 10000, 20000, 5000, 1400 (dynamic)	10, 50, 300, 1100 (dynamic), 2000, 5000	11 inversion times	-	11 inversion times	-
Flip angles (°)	2, 8, 17	25	2, 5, 10, 16	8	1, 10, 19	8 (10)	2 to 14 (5 angles)	45	6	35	7, 16, 37	35	2, 5, 10, 15
No. slices	25	40	25	1-2	36	1-2	-	1	41	1	-	1	-
Slice (mm)	4	5	4.2	8	5	8	3	7	5	5	5	10	5
FoV (mm)	375	230	230	420 × 288.75	350 × 260	420 × 290 (320 × 280)	240 to 260	375	450	380	240	-	-
Resolution (mm)	2.93	1.8	1.8	3.3	0.9	3.3 (1.0)	0.94	2.93	4.69	1.7	1.88	-	-
Dynamic / Static	Dynamic	Dynamic	Dynamic	Static	Static	Static	Static	Dynamic	Dynamic	Static	Static	Static	Static
Temp res. (s)	20	2.3	68	-	-	-	-	30	10	-	-	-	-
Hyperoxia	100% (15L/min)	Carbogen: 95% O_2_	100% (15L/min)	100 % / Carbogen	15 L/min for 10–15 mins	15 L/min for 10–15 mins	100% for 7 mins	100% (15L/min)	100% (15L/min)	100% until end tidal O2 = 70%	100% for 3 min	100% until end tidal O_2_ = 70%	100% until end tidal O_2_ = 70%
Oxygen delivery device	Non-rebreathing circuit with Hudson mask.	Mask	Non-rebreathing mask	Non-rebreathing mask	-	-	Face mask	Non-rebreathing mask	Non-rebreathing Hudson mask via gas blender	Non-rebreathing mask	Non-rebreathing mask	Non-rebreathing mask	Non-rebreathing mask
Coils	Body	Head	Head	Spine and body	Spine and body	Spine and body	Cardiac andendorectal	Body	Body	Body	Head	Head	Head
Duration baseline	24 readings	1 min	11 readings	1 reading	1 reading	1 reading	1 reading	9 readings	12/18 readings	1 reading	1 reading	1 reading	1 reading
Duration oxygen	48 readings	4 min	12 readings	1 reading	1 reading	1 reading	1 reading	-	48 readings	1 reading	1 reading	1 reading	1 reading
OE-MRI duration	32 mins	7 min	26 min	20 s per reading	14 s per image	18 s (10 s) per image	-	-	26 mins	-	-	-	-

FFE, Fast Field Echo; IR, Inversion Recovery; IR-LL, Inversion Recovery Look-Locker; MOLLI, Modified Look-Locker Inversion Recovery; SPGR, Spoiled gradient recalled acquisition in the steady state;VFA, Variable Flip Angle.

**Table 3.** Imaging parameters and setup details for the T_1_ based oxygen-enhanced MR scans for the conference abstracts of human studies

	Panek et al. 2018^44^	Little et al. 2019^45^	Datta et al. 2022^11^	Dubec et al. 2022^54^	McCabe et al. 2022^12^	Prezzi et al. 2022 a^53^	Prezzi et al. 2022 b^53^
Area	Head and neck	Rectum	Cervix	Head and neck	Head and neck	Rectum	Rectum
B_0_	3T	1.5T	1.5T	1.5T	1.5T	3T	3T
Sequence	3D SPGR	3D SPGR	IR prepared 3D SPGR	IR prepared 3D SPGR	Dixon	3D SPGR	MOLLI
TR / TE (ms)	4.5/2.3	12/0.74	2.2/0.66	2.8 to 3.0/0.9 to 1.0	7.3/2.39 & 4.77 ms	4.56/2.04	280.56/1.12
TI (ms)	-	-	100, 500, 1100 (dynamic), 2000, 4300	100, 500, 800, 1100 (dynamic), 4300	-	-	180
Flip angles (°)	3, 16	3, 13, 18	4	6	2, 12	2, 13	35
No. slices	24	25	-	-	72	72	1
Slice (mm)	2.5	4	6	5	2.5	3	8
FoV (mm)	240	375	384	384	200	380 × 309	360 × 307
Resolution (mm)	1.5	2.34	3	3	1.6	1.2	1.4
Dynamic / Static	Dynamic	Dynamic	Dynamic	Dynamic	Static	Static	Static
Temp res. (s)	3	13.9	12	12	-	-	-
Hyperoxia	100%	100%	100%	100%	100% for ≥ 7 mins	100% for ≥ 3 mins	100% for ≥ 3 mins
Oxygen delivery device	Non-rebreathing mask	Non-rebreathing mask	Non-rebreathing mask	Non-rebreathing mask	Non-rebreathing mask	-	-
Coils	Head and neck	-	-	-	Posterior head / anterior flex	-	-
Duration baseline	20 readings	14 readings	25 readings	25 readings	5 readings	6 readings	6 readings
Duration oxygen	210 readings	-	45 readings	45 readings	5 readings	6 readings	6 readings
OE-MRI duration	-	-	19 mins	19 mins	8.6 s per reading	40 s per reading	8.5 s per reading

IR, Inversion Recovery; MOLLI, Modified Look-Locker Inversion Recovery;SPGR, Spoiled gradient recalled acquisition in the steady state.

No imaging parameters were provided for one abstract.56

OE-MRI can be performed statically with T_1_ mapping performed before and after oxygen or dynamically during the switch from air to oxygen (Tables 2 and 3). The duration of hyperoxia delivered before repeat imaging in human studies ranges from 2 to 15 min. For the 6 human studies that provide a dynamic scan duration, the median OE-MRI acquisition time was 22.5 min (range 7–32 min).

#### Oxygen delivery

The oxygen challenge was delivered in the form of 100% oxygen in 35 studies (71.4%) and carbogen (mixture of oxygen and carbon dioxide) in 9 studies (18.4%) with 2 studies using both and 3 unstated. In the human studies, the majority use 100% oxygen (15 studies, 83.3%) with 2 using carbogen (11.1%) and 1 using both. Carbogen has been investigated as an alternative to 100% oxygen with the aim of mitigating the vasoconstrictive effect of hyperoxia with the vasodilative influence of carbon dioxide.^15,16,19,20,22,23,25,33^ However, Winter et al found that varying the carbon dioxide concentration in administered carbogen had no significant effect on altering blood flow during OE-MRI^19^ and Hallac *et al* found similar OE-MRI responses in prostate cancer xenografts with carbogen and 100% oxygen.^16^ It should be noted that in animal experiments,high concentration oxygen is also a crucial component of the anaesthetic process, therefore potentially affecting baseline oxygenation levels compared to awake animals.

The use of an internal quality control point to provide a quantitative assessment of adequate oxygen delivery has been proposed because inadequate oxygen delivery during an OE-MRI scan could result in inappropriate labelling of regions as oxygen challenge refractory. Such control regions have been located in skeletal muscle,^5^ renal cortices,^29^ descending thoracic aorta,^31^, uterine body^11^ and nasal conchae.^55^

## Discussion

Overall, there is strong pre-clinical evidence that OE-MRI can accurately and reliably detect hypoxic regions of solid tumours and monitor how such regions change with anticancer therapies. The evidence base for OE-MRI from clinical studies is, however, much less advanced. Initial results from human trials are promising for the utility of OE-MRI in tumour hypoxia imaging, however, due to the early stage nature of this research all of these studies are single institute trials without prospective power calculations and without standardised data acquisition or analysis methodologies. Further work is required on specific tumour sites in patients to optimise and standardise OE-MRI protocols as well as establish the optimum timing to correlate OE-MRI data to clinically relevant outcomes before validating OE-MRI biomarkers in larger multicentre trials.

Currently, there is no consensus on the optimal imaging sequence to use in OE-MRI. Given the heterogeneous nature of oxygenation within tumours, it is unsurprising that most researchers have opted for three-dimensional acquisitions in order to map the entire tumour volume. Indeed those clinical studies that opted for single slice acquisitions have struggled with co-registering images acquired during treatment with baseline data.^10,52^ The accuracy and precision of T_1_ determination is not equivalent between different methodologies though; VFA, *e.g.* consistently overestimates T_1_ values in the brain.^59,60^ However, as it is the change in T_1_ times that is relevant in OE-MRI, this may mitigate somewhat systematic errors in T_1_ measurement. Work to develop a consensus guideline, such as exists in DCE MRI,^61^ balancing the competing demands of acquisition time, spatial coverage, temporal resolution and measurement accuracy is critical in standardising tumour OE-MRI imaging and allowing for comparison of studies.

If OE-MRI were to be added to routine clinical diagnostic protocols, the duration of the OE-MRI sequence is critical with respect to health resources, patient tolerability and the risk of movement and image quality degradation. The extent and nature of movement during OE-MRI scans will vary depending upon the anatomical area of interest; however, it is clear that appropriate image co-registration techniques are required for robust data analysis.^10,12,31^ Currently, there is a large range in the duration of clinical OE-MRI scans and variations between dynamic imaging and static acquisitions. The optimal duration of the oxygen challenge in patients is not yet proven and the potential benefits of delivering multiple oxygen challenges during one imaging session thus facilitating independent component data analysis techniques has not been proven to outweigh the difficulties of increased scan time.

With regards to the delivery of the oxygen challenge, although there are legitimate concerns regarding the vasoconstrictive effects of hyperoxia confounding the OE-MRI signal, the evidence presented suggests that in practice this is not a significant issue. In addition, inhalation of carbogen gas has been shown to induce unpredictable responses in different organs^62^ as well as not always being well tolerated in humans due to its potential to cause dyspnoea. It therefore seems reasonable for future human studies to use 100% oxygen rather than carbogen, however, the risk of hyperoxic vasoconstriction should be considered. In addition, future studies should continue to use quality control points to provide quantitative evidence of adequate tissue oxygen delivery. The location of such points will depend on the anatomical area imaged, the field of view used and the imaging sequence. Standardisation of such markers will be helpful for future multicentre trials.

Novel methods of OE-MRI data analysis continue to be developed and offer the potential to increase the accuracy of OE-MRI in differentiating regions of tumour hypoxia. Although the use of the perfused hypoxic fraction metric has been successfully applied in clinical OE-MRI studies, it does require a co-registered perfusion assessment which may limit its clinical utility. Alternative approaches such as synchronous BOLD and OE-MRI measurements, independent or principle component data analysis and novel data clustering techniques may ultimately prove to be more effective hypoxia categorisation tools. Further work is required to correlate data clustering approaches to clinically relevant outcomes and to optimise OE-MRI data processing approaches.

The optimal timing of when to perform OE-MRI assessments of tumours in patients undergoing treatment is not yet clear. Baseline metrics of hypoxic fraction may provide a stratification methodology for the utilisation of novel hypoxia activated drugs or novel radiosensitisers in patients more likely to respond to them but the more sensitive application of OE-MRI biomarkers may be in identifying regional hypoxia that is invariant to treatment. The initial patient studies looking at such repeat scans in OE-MRI show that the timing of the reassessments is crucial but this may also be dependent on tumour type and therapy modality and requires further evaluation. In addition, the clinical implication of chronic tumour hypoxia *vs* transient or cycling hypoxia and the potential for OE-MRI to distinguish between these has not been fully explored as yet. Dynamic or repeated oxygen challenge imaging may allow rapid frequency cycling tumour hypoxia characteristics to be elucidated with OE-MRI, whereas repeat assessment on different days is more likely to reveal changes in chronic hypoxia levels.^63^ Separating out these two components of hypoxia may provide a more powerful OE-MRI metric and requires further research.

There are some limitations with this scoping review. Firstly, due to the relatively novel nature of OE-MRI in solid tumours just over 30% of the published articles identified are conference abstracts rather than journal articles. These abstracts have not been through the same level of peer review that journal articles are subject to; however, we felt that it was important to include this grey literature in order to present the full scope of research being performed in this area. Secondly, our inclusion criteria explicitly states that we are interested in studies that quantify oxygen induced T_1_ or R_1_ changes, however, this means at least three studies that assessed changes in T_1_ weighted signal intensity were excluded from the analysis.^64–66^ Finally, we did not search all available medical or scientific databases but focussed on two that we felt were most likely to yield the greatest number of results. Future reviews may benefit from using alternative databases in order to obtain the most comprehensive search results.

In conclusion, there is strong pre-clinical evidence of the utility of OE-MRI in assessing and monitoring tumour hypoxia, however, significant clinical work remains to be completed before OE-MRI-derived biomarkers can be utilised as a routine component of cancer imaging.
