# Supplementary material for: T1 based oxygen-enhanced MRI in tumours; a scoping review of current research
Source: Br J Radiol. 2023 Mar 3;96(1146):20220624. doi: 10.1259/bjr.20220624 (PMC10230402; doi:10.1259/bjr.20220624)
Supplement: Supplementary Material 1. [file bjr.20220624.suppl-01.docx]

**Appendix 1: Search strategy**

Search in PubMed, performed 27^th^ May 2022:

| **#** | **Search Terms** | **Results** |
| --- | --- | --- |
| #1 | (MRI) OR ("magnetic resonance") | 901,366 |
| #2 | (cancer*) OR (tumour*) OR (tumor*) OR (malignan*) | 4,174,778 |
| #3 | ("oxygen enhanced") OR ("tissue oxygen level dependent") OR (“tissue oxygenation level dependent”) OR ("oxygen sensitive") OR ("O2 sensitive") OR (hyperoxi*) | 13,141 |
| #4 | #1 AND #2 AND #3 | 134 |
| #5 | #4 AND English[lang] | 134 |

Search in Web of Science, performed 27^th^ May 2022:

| **#** | **Search Terms** | **Results** |
| --- | --- | --- |
| #1 | (MRI) OR ("magnetic resonance") | 830,038 |
| #2 | (cancer*) OR (tumour*) OR (tumor*) OR (malignan*) | 4,857,728 |
| #3 | ("oxygen enhanced") OR ("tissue oxygen level dependent") OR (“tissue oxygenation level dependent”) OR ("oxygen sensitive") OR ("O2 sensitive") OR (hyperoxi*) | 16,398 |
| #4 | #1 AND #2 AND #3 | 189 |
| #5 | #4 AND English[lang] | 189 |
