# Supplementary material for: T1 based oxygen-enhanced MRI in tumours; a scoping review of current research
Source: Br J Radiol. 2023 Mar 3;96(1146):20220624. doi: 10.1259/bjr.20220624 (PMC10230402; doi:10.1259/bjr.20220624)
Supplement: Supplementary Material 2. [file bjr.20220624.suppl-02.docx]

**Appendix 2: Results of Search Strategy after Full Text Review**

| ***Publication type*** | ***Reference*** | ***Title*** | ***Tumour Model*** |
| --- | --- | --- | --- |
| Journal article | Kinoshita et al. 2000 [(67)](https://paperpile.com/c/R9gCgh/VOiB2) | Preservation of tumour oxygen after hyperbaric oxygenation monitored by magnetic resonance imaging | Animal  *SCC VII (murine)* |
| Journal article | Matsumoto et al. 2006 [(68)](https://paperpile.com/c/R9gCgh/K7eN0) | MR assessment of changes of tumour in response to hyperbaric oxygen treatment | Animal  *SCC (murine)* |
| Journal article | O’Connor et al. 2009 [(5)](https://paperpile.com/c/R9gCgh/cCY1o) | Preliminary Study of Oxygen-Enhanced Longitudinal Relaxation in MRI: A Potential Novel Biomarker of Oxygenation Changes in Solid Tumors | Human  *Ovarian (4 subtypes), HCC, Colon, Gastric adeno, Cervix SCC* |
| Journal article | Winter et al. 2011 [(19)](https://paperpile.com/c/R9gCgh/jh3cO) | Quantitative MRI assessment of VX2 tumour oxygenation changes in response to hyperoxia and hypercapnia | Animal  *VX2 carcinoma (rabbit)* |
| Journal article | Burrell et al. 2013 [(20)](https://paperpile.com/c/R9gCgh/EBYUh) | Exploring ΔR(2) * and ΔR(1) as imaging biomarkers of tumour oxygenation | Animal  *Rat GH3 prolactinomas (murine)*  *Human PC3 prostate (murine)* |
| Journal article | Jordan et al. 2013 ([14)](https://paperpile.com/c/R9gCgh/vu5TS) | Mapping of oxygen by imaging lipids relaxation enhancement: A potential sensitive endogenous MRI contrast to map variations in tissue oxygenation | Animal  *NT2 breast (murine)*  *MDA-MB-231 breast (murine)* |
| Journal article | Remmele et al. 2013 [(21)](https://paperpile.com/c/R9gCgh/3UhqC) | Dynamic and simultaneous MR measurement of R1 and R2*changes during respiratory challenges for the assessment of blood and tissue oxygenation | Human  *Intracranial (metastasis, GBM, lymphoma)* |
| Journal article | Colliez et al. 2014 [(15)](https://paperpile.com/c/R9gCgh/hxjj0) | Qualification of a noninvasive magnetic resonance imaging biomarker to assess tumour oxygenation | Animal  *NT2 breast (murine)*  *MDA-MB-231 breast (murine)* |
| Journal article | Hallac et al. 2014 [(16)](https://paperpile.com/c/R9gCgh/iV1e2) | Correlations of Noninvasive BOLD and TOLD MRI with pO(2) and Relevance to Tumour Radiation Response | Animal  *Dunning R3327-AT1 prostate (rat)* |
| Journal article | Linnik et al. 2014 [(36)](https://paperpile.com/c/R9gCgh/ZOQd9) | Noninvasive Tumour Hypoxia Measurement Using Magnetic Resonance Imaging in Murine U87 Glioma Xenografts and in Patients with Glioblastoma | Mixed  *U87MG human glioma (murine)*  *GBM (human study)* |
| Journal article | Zhao et al. 2015 [(22)](https://paperpile.com/c/R9gCgh/xqoQL) | Dynamic oxygen challenge evaluated by NMR T-1 and T-2* - insights into tumour oxygenation | Animal  *Dunning R3327-AT1 prostate (rat)*  *Dunning R3327-HI prostate (rat)* |
| Journal article | Bane et al. 2016 [(49)](https://paperpile.com/c/R9gCgh/fryov) | Feasibility and reproducibility of BOLD and TOLD measurements in the liver with oxygen and carbogen gas challenge in healthy volunteers and patients with hepatocellular carcinoma | Human  *HCC* |
| Journal article | Beeman et al. 2016 [(23)](https://paperpile.com/c/R9gCgh/XPMfT) | O2-Sensitive MRI Distinguishes Brain Tumour Versus Radiation Necrosis in Murine Models | Animal  *Glioma (murine)* |
| Journal article | Belfatto et al. 2016 [(43)](https://paperpile.com/c/R9gCgh/oXx15) | Tumour radio-sensitivity assessment by means of volume data and magnetic resonance indices measured on prostate tumour bearing rats | Animal  *Dunning R3327-AT1 prostate (rat)* |
| Journal article | Cao-Pham et al. 2016 [(42)](https://paperpile.com/c/R9gCgh/JmAqD) | Monitoring Tumour Response to Carbogen Breathing by Oxygen-Sensitive Magnetic Resonance Parameters to Predict the Outcome of Radiation Therapy: A Preclinical Study | Animal  *Rhabdomyosarcoma (rat)*  *9L-glioma (rat)* |
| Journal article | O’Connor et al. 2016 [(17)](https://paperpile.com/c/R9gCgh/NrSRy) | Oxygen-Enhanced MRI Accurately Identifies, Quantifies, and Maps Tumour Hypoxia in Preclinical Cancer Models | Animal  *786-0 renal (murine)*  *786-0-R renal (murine)*  *SW-620 colorectal (murine)* |
| Journal article | Rich and Seshadri 2016 [(24)](https://paperpile.com/c/R9gCgh/LPPz3) | Photoacoustic monitoring of tumour and normal tissue response to radiation | Animal  *HNSCC (murine)* |
| Journal article | White et al. 2016 [(39)](https://paperpile.com/c/R9gCgh/ifplo) | Developing Oxygen-Enhanced Magnetic Resonance Imaging as a Prognostic Biomarker of Radiation Response | Animal  *Dunning R3327-AT1 prostate (rat)* |
| Journal article | Cao-Pham 2017 [(25)](https://paperpile.com/c/R9gCgh/WaV2e) | Combined endogenous MR biomarkers to predict basal tumour oxygenation and response to hyperoxic challenge | Animal  *Rhabdomyosarcoma (rat)*  *9L-glioma (rat)* |
| Journal article | Hectors et al. 2017 [(55)](https://paperpile.com/c/R9gCgh/iAtbt) | Quantification of hepatocellular carcinoma heterogeneity with multiparametric magnetic resonance imaging | Human  *HCC* |
| Journal article | Zhou, Hallac et al. 2017 [(50)](https://paperpile.com/c/R9gCgh/77OTV) | Incorporating Oxygen-Enhanced MRI into Multi-Parametric Assessment of Human Prostate Cancer | Human  *Prostate* |
| Journal article | Zhou, Zhang et al. 2017 [(27)](https://paperpile.com/c/R9gCgh/z0E8b) | Tumour physiological changes during hypofractionated stereotactic body radiation therapy assessed using multi-parametric magnetic resonance imaging | Animal  *A549 human NSCLC (rat)* |
| Journal article | Baker et al. 2018 [(69)](https://paperpile.com/c/R9gCgh/zRBth) | Evaluating Imaging Biomarkers of Acquired Resistance to Targeted EGFR Therapy in Xenograft Models of Human Head and Neck Squamous Cell Carcinoma | Animal  *CAL^R^ HNSCC (murine)*  *CAL^S^ HNSCC (murine)* |
| Journal article | Featherstone, O’Connor et al. 2018 [(28)](https://paperpile.com/c/R9gCgh/fn86R) | Data-Driven Mapping of Hypoxia-Related Tumour Heterogeneity Using DCE-MRI and OE-MRI | Animal  *U87 glioma (murine)*  *Calu6 NSCLC (murine)* |
| Journal article | Little et al. 2018 [(29)](https://paperpile.com/c/R9gCgh/BZNYu) | Mapping Hypoxia in Renal Carcinoma with Oxygen-enhanced MRI: Comparison with Intrinsic Susceptibility MRI and Pathology | Mixed  *786-0-R renal (murine)*  *RCC (human)* |
| Journal article | Moosvi et al. 2019 [(38)](https://paperpile.com/c/R9gCgh/ehVbe) | Fast and sensitive dynamic oxygen-enhanced MRI with a cycling gas challenge and independent component analysis | Animal  *SCC VII (murine)*  *HCT-116 colorectal (murine)*  *SKOV3 ovarian (murine)*  *BT-474 breast (murine)* |
| Journal article | Salem et al. 2019 [(31)](https://paperpile.com/c/R9gCgh/6roq6) | Oxygen-enhanced MRI Is Feasible, Repeatable, and Detects Radiotherapy-induced Change in Hypoxia in Xenograft Models and in Patients with Non-small Cell Lung Cancer | Mixed  *U87 glioma (murine)*  *Calu6 NSCLC (murine)*  *NSCLC (human)* |
| Journal article | Yang et al. 2019 [(47)](https://paperpile.com/c/R9gCgh/pDTI8) | Oxygen-sensitive MRI assessment of tumour response to hypoxic gas breathing challenge | Animal  *13762NF breast (rat)* |
| Journal article | Zhou et al. 2019 [(18)](https://paperpile.com/c/R9gCgh/NFuoB) | Examining correlations of oxygen sensitive MRI (BOLD/TOLD) with [(18)F]FMISO PET in rat prostate tumors | Animal  *Dunning R3327-AT1 prostate (rat)* |
| Journal article | Bluemke et al. 2020 [(51)](https://paperpile.com/c/R9gCgh/VXFMX) | Oxygen-enhanced MRI MOLLI T1 mapping during chemoradiotherapy in anal squamous cell carcinoma | Human  *Anal SCC* |
| Journal article | Qian et al. 2020 [(52)](https://paperpile.com/c/R9gCgh/4dQWi) | In vivo Monitoring of Oxygen Levels in Human Brain Tumour Between Fractionated Radiotherapy Using Oxygen-enhanced MR Imaging | Human  *Brain metastases* |
| Journal article | Waschkies et al. 2020 [(33)](https://paperpile.com/c/R9gCgh/XxARF) | Tumour grafts grown on the chicken chorioallantoic membrane are distinctively characterized by MRI under functional gas challenge | Animal  *A549 human NSCLC (CAM)*  *MC-38 murine colon (CAM)* |
| Journal article | Arai et al. 2021 [(41)](https://paperpile.com/c/R9gCgh/Wo1l1) | Oxygen-Sensitive MRI: A Predictive Imaging Biomarker for Tumour Radiation Response? | Animal  *Dunning R3327-AT1 prostate (rat)* |
| Journal article | Bluemke et al. 2022 [(10)](https://paperpile.com/c/R9gCgh/FP28Y) | Using Variable Flip Angle (VFA) and Modified Look-Locker Inversion Recovery (MOLI) T1 mapping in clinical OE-MRI | Human  *HNSCC* |
| Conference abstract | Pacheco-Torres et al. 2009 [(13)](https://paperpile.com/c/R9gCgh/eaCXg) | Evaluation of lung tumour oxygenation using FREDOM and TOLD | Animal  *A549 human NSCLC (rat)* |
| Conference abstract | Zhou et al. 2010 [(70)](https://paperpile.com/c/R9gCgh/Mq47e) | Integrated MRI approaches to interrogate tumour oxygenation and vascular perfusion of orthotopic brain tumors in a mouse model | Animal  *U87 glioma (murine)* |
| Conference abstract | Zhou et al. 2016 [(56)](https://paperpile.com/c/R9gCgh/vKhzk) | TOLD MRI Validation of Reversal of Tumour Hypoxia in Glioblastoma with a Novel Oxygen Therapeutic | Human  *GBM* |
| Conference abstract | Little et al. 2017 [(40)](https://paperpile.com/c/R9gCgh/Nx4ep) | In vivo OE-MRI quantification and mapping of response to hypoxia modifying drugs Banoxantrone and Atovaquone in Calu6 xenografts | Animal  *Calu6 NSCLC (murine)* |
| Conference abstract | Zhou et al. 2017 [(26)](https://paperpile.com/c/R9gCgh/kOJEa) | Evaluation of tumour oxygenation following radiation and PS-targeting antibody therapy in an orthotopic lung cancer model | Animal  *A549 human NSCLC (rat)* |
| Conference abstract | Li et al. 2018 [(37)](https://paperpile.com/c/R9gCgh/kjYZM) | Assessment of Tumour Hypoxia Using Tissue Oxygen Level Dependent in a Rabbit VX2 Liver Tumour model | Animal  *VX2 carcinoma (rabbit)* |
| Conference abstract | Panek et al. 2018 [(44)](https://paperpile.com/c/R9gCgh/LQOyR) | Oxygen-Enhanced MRI for the Detection of Hypoxia in Patients with Head and Neck Cancer | Human  *HNSCC* |
| Conference abstract | Little et al. 2019 [(45)](https://paperpile.com/c/R9gCgh/EZlZG) | OE-MRI, DCE-MRI and DWI provide complementary response evaluation in patients with rectal cancer treated with chemoradiotherapy | Human  *Rectal* |
| Conference abstract | Lepicard et al. 2020 [(32)](https://paperpile.com/c/R9gCgh/fhW7V) | Imaging hypoxia in head and neck cancer xenografts with oxygen-enhanced MRI | Animal  *CAL^R^ HNSCC (murine)*  *CAL^S^ HNSCC (murine)*  *LICR-LON-HN5 HNSCC (murine)*  *786-O-R renal (murine)* |
| Conference abstract | Boult et al. 2022 [(34)](https://paperpile.com/c/R9gCgh/B1vsA) | Characterising hypoxia in rhabdomyosarcoma xenografts with oxygen-enhanced MRI | Animal  *RH41 RMS (murine)*  *RD RMS (murine)*  *RH30 RMS (murine)* |
| Conference abstract | Datta et al. 2022 [(11)](https://paperpile.com/c/R9gCgh/We2os) | Quantifying and mapping hypoxia modification in patients with uterine cervical cancer using oxygen-enhanced MRI | Human  *SCC cervix* |
| Conference abstract | Dubec et al. 2022 [(54)](https://paperpile.com/c/R9gCgh/PVnyx) | First in-human technique translation of OE-MRI for hypoxia imaging onto an MR Linac system in patients with head and neck cancer | Human  *HNSCC* |
| Conference abstract | McCabe et al. 2022 [(12)](https://paperpile.com/c/R9gCgh/3bI3m) | Oxygen induced T1 changes in head and neck anatomical structures | Human  *HNSCC* |
| Conference abstract | Prezzi et al. 2022 [(53)](https://paperpile.com/c/R9gCgh/vK33B) | Feasibility and repeatability of oxygen-enhanced T1 measurements in primary colorectal cancer: a prospective study in 22 patients | Human  *Colorectal* |
| Conference abstract | Roy et al. 2022 [(35)](https://paperpile.com/c/R9gCgh/QQj6p) | Imaging hypoxia in murine oral cavity squamous cell carcinomas with oxygen-enhanced MRI | Animal  *MOC1 OSCC (murine)*  *MOC2 OSCC (murine)* |

*Abbreviations: CAM - chorioallantoic membrane, GBM – glioblastoma multiforme, HCC – hepatocellular carcinoma, HNSCC – head and neck squamous cell carcinoma, NSCLC – non-small cell lung cancer, OSCC – oral cavity squamous cell carcinoma, RCC – renal cell carcinoma, RMS – Rhabdomyosarcoma, SCC – squamous cell carcinoma*
